# Supplementary figures and images for: Why Early Tactile Speech Aids May Have Failed: No Perceptual Integration of Tactile and Auditory Signals
Source: Front Psychol. 2018 May 23;9:767. doi: 10.3389/fpsyg.2018.00767 (PMC5974558; doi:10.3389/fpsyg.2018.00767)

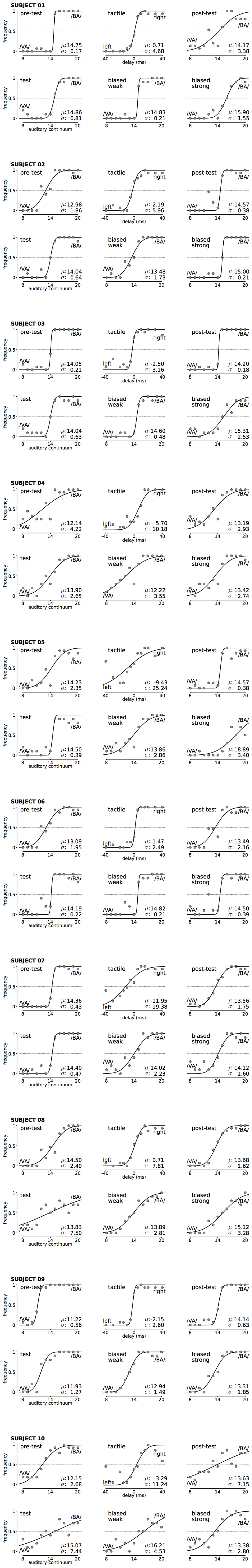

Supplement: FIGURE S1 — Audio and tactile psychometric functions fitted to the data of the 10 subjects. For the audio graphs, each data point corresponds to 15 measurements. For the tactile graph (top, middle), each data point corresponds to 15 measurements. The means μ of each function are taken to be the PSE for that subject for that condition, and σ indicates the slope. [file Image_1.JPEG]
